# Strain Specific Phage Treatment for *Staphylococcus aureus* Infection Is Influenced by Host Immunity and Site of Infection

**DOI:** 10.1371/journal.pone.0124280

**Published:** 2015-04-24

**Authors:** Nathan B. Pincus, Jensen D. Reckhow, Danial Saleem, Momodou L. Jammeh, Sandip K. Datta, Ian A. Myles

**Affiliations:** Bacterial Pathogenesis Unit, Laboratory of Clinical Infectious Diseases, National Institute of Allergy and Infectious Diseases, National Institutes of Health, Bethesda, Maryland, United States of America; Leibniz-Institute DSMZ, GERMANY

## Abstract

The response to multi-drug resistant bacterial infections must be a global priority. While mounting resistance threatens to create what the World Health Organization has termed a “post-antibiotic era”, the recent discovery that antibiotic use may adversely impact the microbiome adds further urgency to the need for new developmental approaches for anti-pathogen treatments. Methicillin-resistant *Staphylococcus aureus* (MRSA), in particular, has declared itself a serious threat within the United States and abroad. A potential solution to the problem of antibiotic resistance may not entail looking to the future for completely novel treatments, but instead looking into our history of bacteriophage therapy. This study aimed to test the efficacy, safety, and commercial viability of the use of phages to treat *Staphylococcus aureus* infections using the commercially available phage SATA-8505. We found that SATA-8505 effectively controls *S*. *aureus* growth and reduces bacterial viability both in vitro and in a skin infection mouse model. However, this killing effect was not observed when phage was cultured in the presence of human whole blood. SATA-8505 did not induce inflammatory responses in peripheral blood mononuclear cultures. However, phage did induce IFN gamma production in primary human keratinocyte cultures and induced inflammatory responses in our mouse models, particularly in a mouse model of chronic granulomatous disease. Our findings support the potential efficacy of phage therapy, although regulatory and market factors may limit its wider investigation and use.

## Introduction

Resistance to antibiotics is one of the most significant challenges in healthcare today. While the incidence of antibiotic resistance to *Staphylococcus aureus* has increased over the nearly nine decades since Alexander Fleming observed the treatment potential of *Penicillium notatum*, the discovery and development of new antibiotics has slowed to a trickle [1–3]. Without development of new treatment modalities, there is a very real threat of approaching what the World Health Organization (WHO) termed a “post-antibiotic era”, where the treatments we have relied on are simply no longer effective and we are left unarmed in the unending silent war against disease [2,3]. Methicillin-resistant *Staphylococcus aureus* (MRSA) has been declared a serious threat by the Centers for Disease Control and Prevention (CDC), causing 80,461 serious infections and 11,285 deaths in the US in 2011 alone [4]. Additionally, the emergence of vancomycin resistant or intermediate *Staphylococcus aureus* (VRSA or VISA) threatens to cause infections that have few treatment options [1]. The rising problem of antibiotic resistance in bacterial infections, and *S*. *aureus* infections in particular, creates a pressing need for research into alternative methods of treatment.

A solution to the problem of antibiotic resistance may not entail discovering completely novel treatments, but instead looking back into history, to bacteriophage therapy. Phage therapy harnesses the killing power of bacteria’s natural predators, lytic bacteriophages, to fight disease [1,5,6]. The concept of phage therapy predates the use of conventional antibiotics, initially put forth by Felix d’Herelle, co-discoverer of phages, in the early 1920’s [5–7]. Although this was followed by an initial boom of interest and research, phage therapy faded from use following the discovery of antibiotics [1,5–7]. However, both phage therapy and research have continued in Eastern Europe and former Soviet states, reporting success in treating a variety of bacterial infections, including those by *S*. *aureus* [1,6]. The rising problem of antibiotic resistance and the need for alternative treatments have led to a resurgence of interest in phage therapy. However, many studies to date lack adequate controls and are poorly designed; additional research is needed to substantiate the safety and efficacy of these treatments before they can be integrated into standard clinical care [1,6,7]. A current pilot clinical trial in Belgium is examining the use of topical application of *S*. *aureus* and *P*. *aeruginosa* phages to treat burn wound infections [1,8]. In addition, staphylococcal phages have other potential healthcare uses, such as application to catheter material to control biofilm formation [9] or in hand wash solutions to lower CFUs of *S*. *aureus* on the skin of healthcare workers [10].

This study aimed to investigate the efficacy, safety, and commercial viability of the use of phages to treat *Staphylococcus aureus* infections. *S*. *aureus* bacteriophage SATA-8505 (ATCC PTA-9476) was examined for its ability to prevent and treat infections with clinically relevant MRSA strain USA300 *in vitro* in human cells and *in vivo* in mice. SATA-8505 was selected as a model phage to assess efficacy for treatment of USA300 infection because of its commercial availability and patented claims of activity against USA300 *in vitro* with classifications 435/235.1 (USA), C12P1/06 (International), C12N7/00, C12N2795/00021 (Cooperative), and C12N7/00 (European) (Patent no: US 7,745,194 B2)[11].

## Materials and Methods

### Materials

Chemical reagents were purchased from Sigma Chemical Company, St. Louis, MO. Blood aglates were from Thermo Scientific, Dubuque, IA. Tryptic Soy Broth and Brain Heart Infusion media/agar were from General Laboratory Products, Yorkville, IL. Fetal Bovine Serum was from Thermo Scientific, Dubuque, IA.

*Mice*. All experiments were approved and monitored by the Institutional Animal Care and Use Committee (IACUC) for the National Institutes of Health. Wild type C57BL/6 mice and B6.129S6-*Cybb* ^*tm1Din*^/J mice, a model for X-linked chronic granulomatous disease (CGD), were purchased from The Jackson Laboratory, Bar Harbor, ME. Mice were 7–12 weeks old during the course of the experiments and were age- and gender-matched within each experiment. All experiments were done in compliance with the guidelines of the NIAID Institutional Animal Care and Use Committee.

### Bacterial and phage strains

USA300 LAC strain of MRSA was a gift from F. DeLeo (Rocky Mountain Labs, NIAID). SATA-8505 bacteriophage, which has been patented for its reported activity against USA300 *in vitro* (Patent no: US 7,745,194 B2)[11], was obtained from ATCC (ATCC PTA-9476). *S*. *aureus* USA100 strains 71080 (NR-46418, VRS8) and 626 (NR-4668) were provided by the Network on Antimicrobial Resistance in *Staphylococcus aureus* (NARSA) for distribution by BEI Resources, NIAID, NIH.

### Phage propagation and selection for efficacy

SATA-8505 was propagated by inoculating 10 mL BHI (Thermo Fisher Scientific) with 100μL of SATA-8505 stock and 100μL of an overnight culture (ONC) of USA300 and incubating at 37°C overnight with shaking. The resulting lysate was centrifuged at 6000g for 12 minutes, the supernatant put through a 0.22 μm filter, and stored at 4°C until use. Plaque forming units (PFUs) of phage were determined using the standard double agar overlay method [12]. Serial dilutions of SATA-8505 stock (100μL) and 100μL of USA300 ONC were mixed into 4 mL molten BHI top agar (0.5% agar) and poured over BHI plates. Plates were incubated overnight at 37°C and the resulting plaques counted. In order to select for SATA-8505 virions against USA300, plaques in the top agar were resuspended in 5mL TSB, centrifuged at 6000g for 15 minutes, the supernatant put through a 0.22 μm filter, and the resulting phage stock was used in subsequent propagations. Experiments using alternate strains of *S*. *aureus* were conducted in an identical manner.

### Phage viability assay

A known concentration of SATA-8505 was divided into 2mL aliquots and stored in the following conditions: room temperature, -80°C, lyophilized then room temperature, lyophilized then 4°C. Phage counts were determined via the top agar method at indicated time points. In addition, the viability of phage stock stored at 4°C was routinely monitored via the double agar overlay method.

### Phage killing kinetics of Staphylococcus in media and whole blood

Approximately 10^6^ CFUs of mid-exponential growth phase USA300 were added to 5 mL aliquots of BHI or TSB with SATA-8505 at varying MOI. Samples were incubated with shaking at 37°C, with bacterial concentration over time determined by plating on BHI (BD Biosciences) or blood (Thermo Fisher Scientific) agar. For the whole blood assay, 10mL blood from normal donors and/or CGD patients was obtained from the NIH Clinical Center Department of Transfusion Medicine and was added to blood culture bottles containing 30mL of TSB (Thermo Fisher Scientific) with SATA-8505 at varying MOI. In experiments using blood, control blood culture vials were injected with an equal volume of Hanks Buffered Salt Solution (HBSS) (Thermo Fisher Scientific). All human samples were obtained under permission of the Institutional Review Board (IRB) for the National Institute of Allergy and Infectious Disease (NIAID) and the National Institutes of Health (NIH) Clinical Center. All participants provided their written consent to the research protocol and IRB consent was obtained prior to blood collection.

### Cytokine response to phage exposure

Keratinocyte cultures were derived as previously described [13]. After cells reached confluence in a 6-well plate (BD Falcon, Bedford, MA), phage was added in escalating doses up to 10^10^ PFU/culture. Supernatants were harvested at 24 hours and analyzed using multiplex (BIO-RAD, Hercules, CA). Peripheral blood mono-nuclear cells (PBMC) were isolated using standard Ficol gradient centrifugation (GE Healthcare Life Sciences, Pittsburgh, PA). Cells were suspended at 2x10^6^/mL in culture media of DMEM (Gibco Invitrogen, Carlsbad, CA), 10% FBS, Hepes buffer (Thermo Scientific), non-essential amino acids (Gibco), penicillin/streptomycin (Gibco), sodium pyruvate (Gibco), and 2-mercapto-ethanol (Gibco). Supernatants were harvested after 72 hours and analyzed using multiplex (BIO-RAD, Hercules, CA).

### Skin infection model

Mice were inoculated subcutaneously with 10^7^ CFU *S*. *aureus*, and skin lesions assessed as previously described [13–15]. The route of phage delivery has a clear effect on the outcomes of phage therapy, with systemic delivery the most efficacious, even in the case of local infections [7]. When treating with phage, intraperitoneal injections were performed immediately prior to MRSA inoculation with either 10^7^ or 10^9^ PFU of SATA-8505 for MOI of 1 or 100. PFU/lesion concentrations were determined by homogenizing a 3mm diameter punch biopsy of the skin lesion in 0.5mL of HBSS, then adding the entire volume to in the double agar overlay method as above. Diluent treatment involved injection with supernatants from overnight USA300 cultures that had been pelleted and filter sterilized in a manner identical to the phage cultures.

### Statistics

Means were compared using two-tailed unpaired t test, or ANOVA with Bonferroni adjustment for comparison of multiple samples, with Prism software (GraphPad, San Diego, CA). ns = not significant, * = p <0.05, ** = p<0.01, *** = p<0.001, **** = p<0.0001.

## Results

### SATA 8505 Effectively Kills USA 300 and Reduces its Viability in vitro

**Fig 1:**
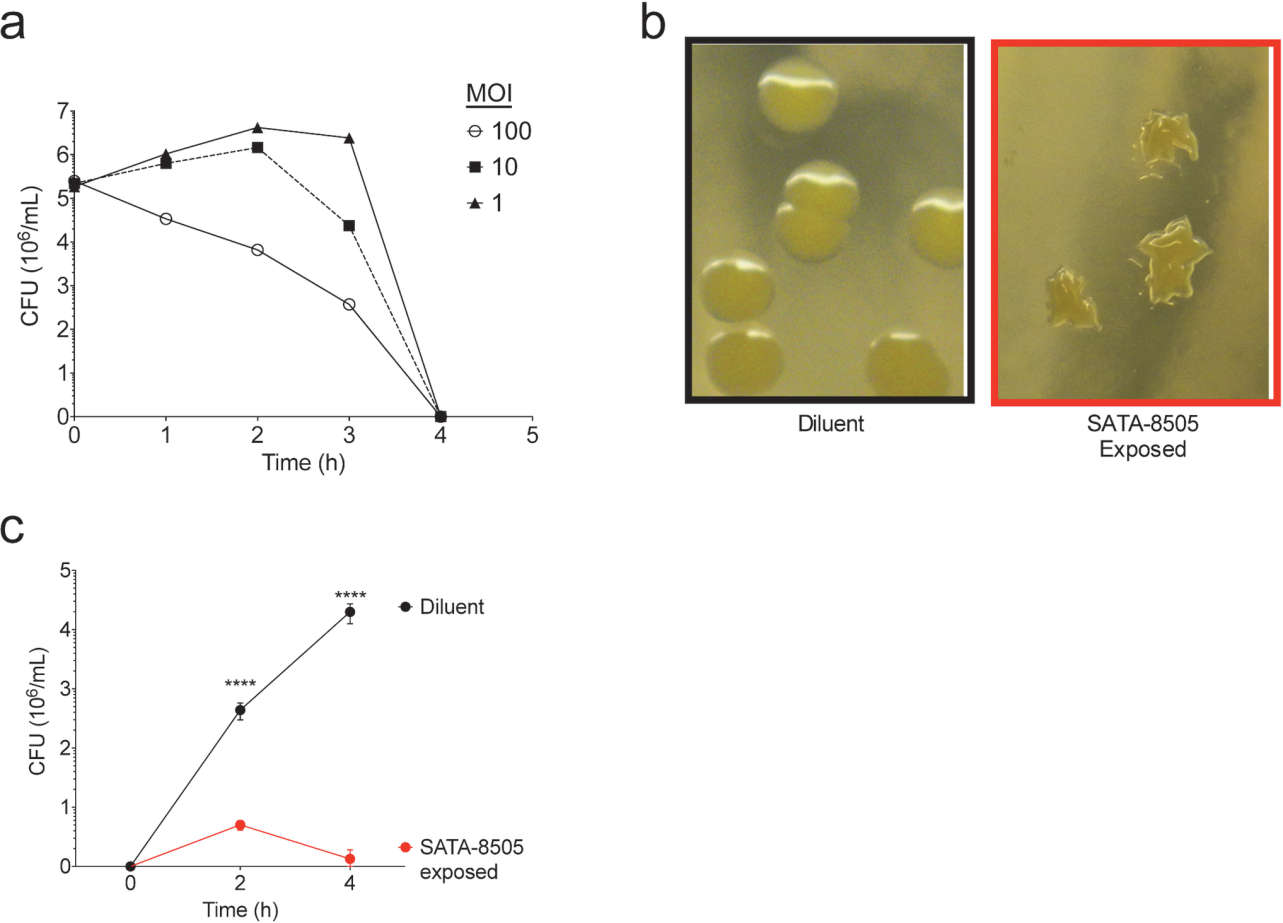
SATA 8505 Effectively Kills USA 300 and Reduces its Viability in vitro. (a) Average colony forming unit (CFU) counts for USA300 cultured with SATA-8505 for up to four hours at ratios of *S*. *aureus*:Phage of 1:1, 1:10, or 1:100 (MOI 1, 10, 100 respectively). (b) Images of surviving colony morphology of USA300 grown in TSB after exposure to BHI (diluent) or SATA-8505. (c) Regrowth of surviving colonies pictured in panel b, *S*. *aureus* grown in TSB after prior exposure to BHI (diluent) or SATA-8505 run in triplicate culture. Data shown are representative of 3 or more independent experiments and displayed as mean + s.e.m. **** = p<0.0001.

USA300 is the leading cause of community-associated MRSA infections in the United States and is increasingly becoming multi-drug resistant [16], causing a great need for alternative treatments. To verify and further quantify the effectiveness of SATA-8505 killing against USA300, we infected 10^6^ colony-forming units (CFU) of mid-exponential growth phase USA300 in Tryptic Soy Broth (TSB) with varying amounts of phage. We then recorded the CFU/mL of bacteria at various time points after phage infection. The entire bacterial cultures were completely killed by phage infection at MOI’s of 1,10, and 100 at 4 hours after inoculation (Fig 1A). Plating earlier time points of the phage-infected culture revealed colonies of MRSA that did grow but showed abnormal colony morphology and were incapable of sustaining growth beyond four hours upon repeat culture in broth (Fig 1B and 1C). Colonies derived after two hours of this repeat culture continued to show abnormal morphology and supernatant from the attempted re-growth cleared subsequent SA cultures, indicating production of phage from the colonies with abnormal morphology (not shown). These results confirm the ability of SATA-8505 to infect and kill USA300.

### SATA-8505 Improves MRSA Skin Infection at Low Multiplicity of Infection (MOI)

**Fig 2:**
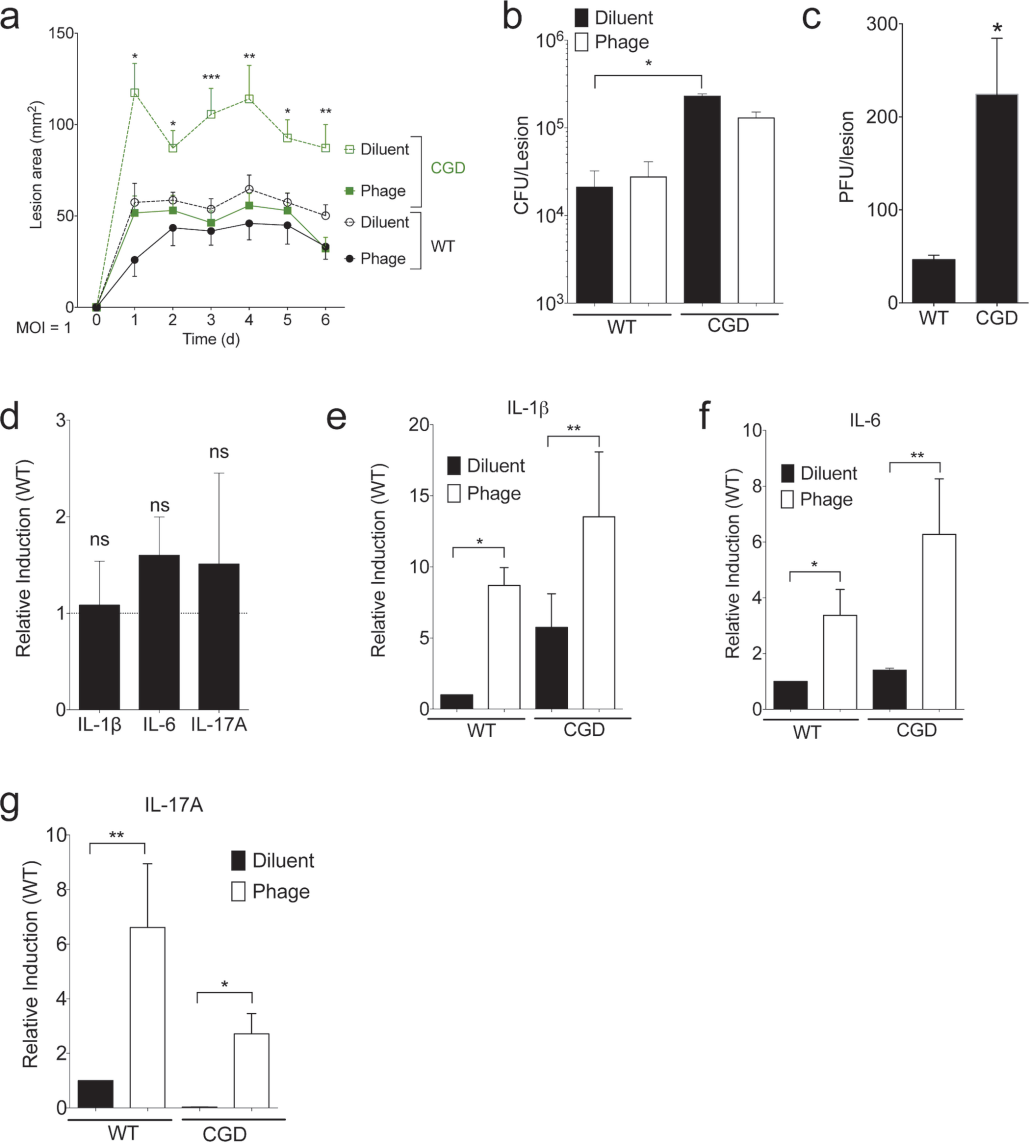
SATA-8505 Improves MRSA Skin Infection at Low MOI. Mice were injected intraperitoneally (I.P.) with 10^7^ plaque-forming units (PFU) of SATA-8505 immediately prior to subcutaneous injections of 10^7^ CFU of USA300. (a) Lesion size progression over following six days. On day 6, skin biopsies were homogenized for culture, total bacterial (b) and phage burden (c) were calculated for individual mice. (d) mRNA transcript levels for IL-1ß, IL-6, and IL-17A in individual CGD mice relative to wild type controls, standardized to GAPDH. (e-g) mRNA transcript levels for IL-1ß, IL-6, and IL-17A in CGD and wild type mice with and without SATA-8505 treatment relative to wild type controls injected with diluent, standardized to GAPDH. Data shown are representative of 2–3 independent experiments using 5 or more mice per group, and displayed as mean + s.e.m. Differences were calculated by ANOVA with Bonferroni correction and depict differences from diluent treated wild type unless otherwise noted. ns = not significant, * = p <0.05, ** = p<0.01.

Phages against *S*. *aureus* have been shown to be effective at killing a multitude of *S*. *aureus* strains *in vitro* [10,17,18], and, unlike antibiotics, have the capacity to rapidly evolve during an infection to overcome developing resistance [10]. Furthermore, phages have shown promise in protecting against *S*. *aureus* sepsis [19–21] and have been beneficial in treating staphylococcal skin and soft tissue infections in mice [20,22]. Therefore, after demonstrating the *in vitro* effectiveness of SATA-8505 at clearing USA300, we evaluated the potential of this specific phage as an antibiotic agent against USA300 *in vivo*. To do this, we used wild type and immunodeficient CGD mice, which are susceptible to *S*. *aureus* due to their dysfunctional neutrophil response. The mice were inoculated with 10^7^ bacteria subcutaneously immediately after intraperitoneal injection of phage. Skin lesion size was quantitatively measured for 6 days after inoculation. Mice treated with phage at a MOI of 1 (1 phage virion to 1 CFU bacteria) showed smaller skin lesion sizes in both wild type and CGD mice as compared to diluent treated controls (Fig 2A). USA300 secretes a variety of virulence factors [23] and thus our phage cultures could stimulate responses due to the presence of these *S*. *aureus* products; to control for this effect, all dilutions and diluent treatments were performed using supernatant from an overnight USA300 culture that had been pelleted and filter sterilized in a manner identical to the phage cultures.

CGD mice had larger lesion sizes than wild type mice in a skin infection model of *S*. *aureus*, a novel and somewhat unexpected finding given the lack of cutaneous *S*. *aureus* infections in patients with CGD, who typically get *S*. *aureus* infections in deep-seated tissues such as the liver [24]. While CGD mice had a greater CFU burden than did wild type, phage treatment failed to significantly alter bacteria counts in either strain (Fig 2B). CGD mice also had higher plaque-forming units (PFU) of SATA-8505 in their lesions (Fig 2C). Despite having slightly larger lesion sizes and bacterial burden during infection (Fig 2A and 2B), CGD mice did not have significant differences in their mRNA levels for IL-1ß, IL-6, or IL-17A (Fig 2D), consistent with their immunodeficiency reflecting a defect in neutrophil function rather than cytokine production [25,26]. While phage treatment reduced lesion size (Fig 2A), it increased lesional inflammatory cytokines in both wild type and CGD mice (Fig 2E–2G). The isolated reduction of lesion size without effects on bacterial burden and cytokine responses suggests that phage therapy at this dose may be inhibiting the ability of bacteria to inflict toxin-mediated dermonecrosis without dramatically affecting viability [27].

**Fig 3:**
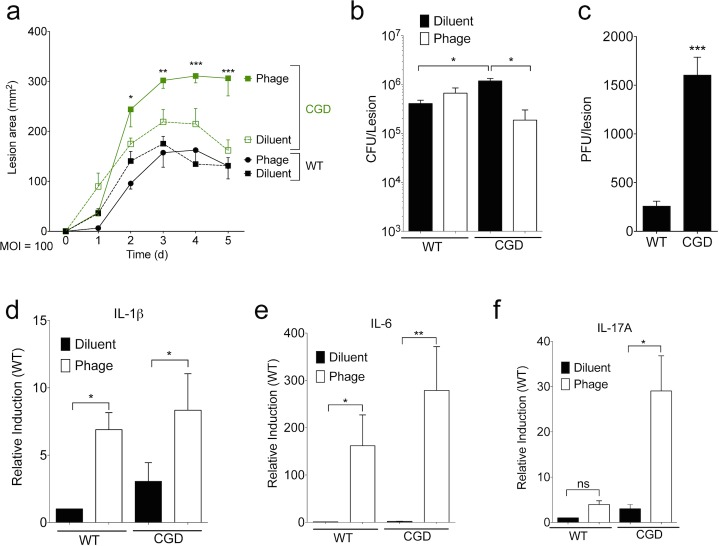
SATA-8505 Fails to Improve MRSA Skin Infection at High MOI. Lesion size (a), CFU (b), PFU (c), and transcript data (d-f) for mice injected with 10^9^ plaque-forming units (PFU) of SATA-8505 immediately prior to subcutaneous injections of 10^7^ CFU of USA300 processed in an identical manner as MOI of 1 experiments. Data shown are representative of 2–3 independent experiments using 5 or more mice per group, and displayed as mean + s.e.m. Differences were calculated by ANOVA with Bonferroni correction and depict differences from diluent treated wild type unless otherwise noted. ns = not significant, * = p <0.05, ** = p<0.01, *** = p<0.001.

Given these results, we hypothesized that increasing the dosage of the bacteriophage would result in greater bacterial clearance. Inoculation of mice with staphylococcal bacteriophage K at doses as high as 2 x 10^11^ PFU was shown to have no effects on physical condition or survival over one month and no effects on organ pathology after 14 days [19]. Accordingly, we increased the dose of phage to an MOI 100 while maintaining a 10^7^ CFU inoculation. CGD mice again had larger lesions and higher bacterial counts than wild type, and the higher MOI phage treatment significantly increased lesion size and reduced bacterial burden in CGD mice (Fig 3A and 3B). CGD mice had significantly higher amounts of virus extracted from their skin lesions than wild type mice (Fig 3C). High dose phage treatment led to increased lesional transcript levels of IL-1ß, IL-6, and IL-17A, particularly in the CGD mice (Fig 3D–3F). Of note, interferon gamma (IFN gamma) was not induced at either MOI treatment (not shown). In these studies at a high phage MOI, phage effects on lesional bacterial burden were only apparent in the setting of CGD and its ineffective neutrophil response. Furthermore, phage therapy induced a vigorous inflammatory response that may have contributed to the increased lesion size despite better bacterial control. Taken together, these studies suggest a complex balance between direct bactericidal activity and induction of an inflammatory response that may contribute to the ultimate efficacy of phage therapy.

### SATA-8505 Induces Interferon Gamma in Primary Human Keratinocytes

**Fig 4:**
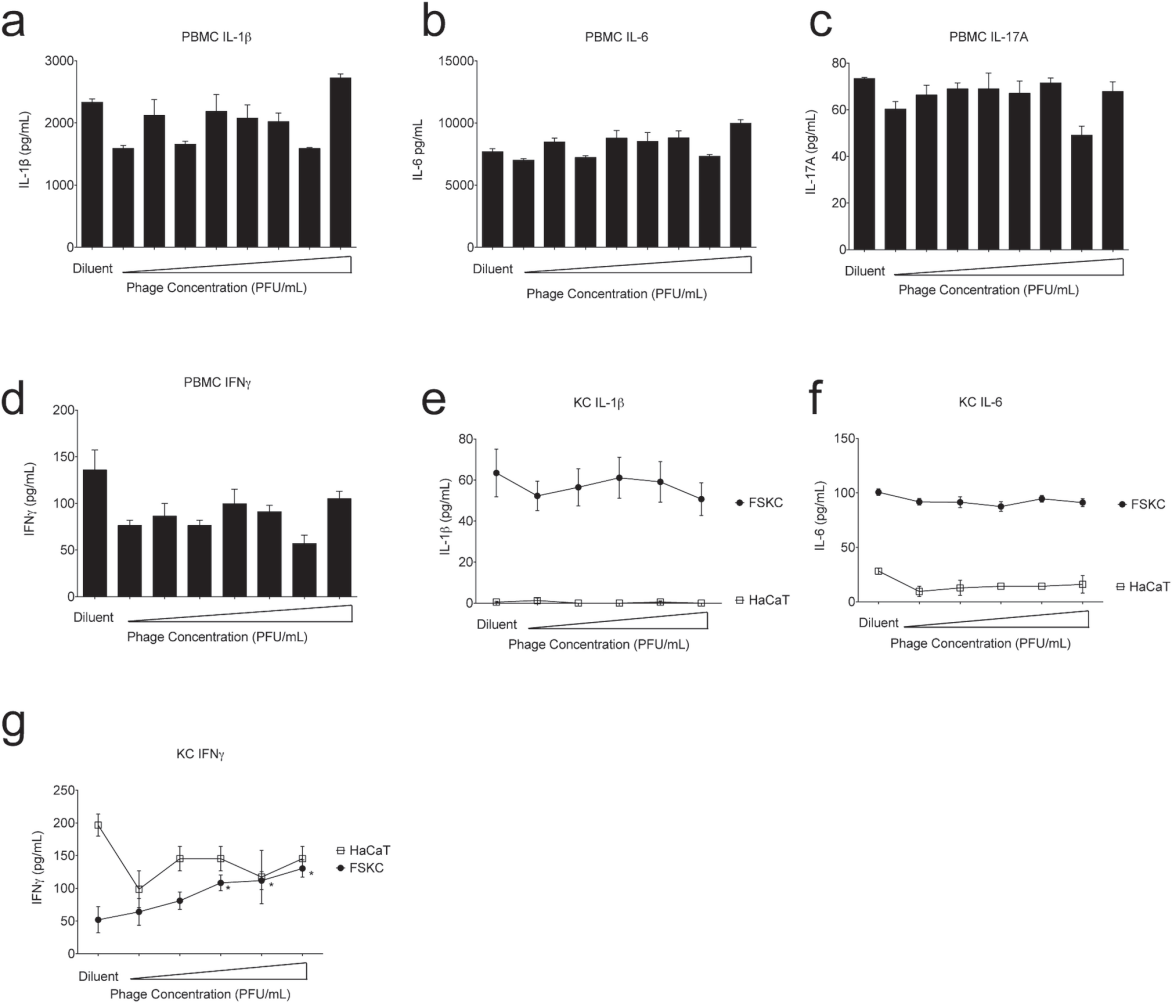
SATA-8505 Induces Interferon Gamma in Primary Human Keratinocytes. Human peripheral mononuclear cells (PBMC) were cultured in triplicate at 2^6^/mL with SATA-8505 at 1 PFU/mL to 10^8^ PFU/mL at ten-fold increments or diluent derived from the supernatant of an overnight culture of SA that had been pelleted and filter sterilized in a manner similar to the phage-containing media. At 72 hours supernatants were harvested and analyzed for IL-1ß (a), IL-6 (b), IL-17A (c), and IFN gamma (d). Human keratinocytes from primary foreskins (foreskin keratinocytes; FSKC) or the HaCaT cell line were cultured to confluence on 6-well plates and incubated in triplicate with SATA-8505 at 10^4^ PFU/mL to 10^8^ PFU/mL at ten-fold increments or TSB diluent. At 24 hours supernatants were harvested and analyzed for IL-1ß (e), IL-6 (f), and IFN gamma (g). Phage for all experiments was diluted in TSB from overnight culture of USA300 that was centrifuged at 5000rpm for 12 minutes and filter-sterilized through a 0.44 micrometer filter. Data shown are representative of 3 independent experiments using 3 different healthy volunteers (a-d) or a pool of 5 or more foreskin samples (e-g) and displayed as mean + s.e.m. * = p <0.05.

Given our findings that phage treatment could increase inflammatory responses in mice, we next investigated if similar responses could be seen in human cells. Phage therapy would only be feasible in the absence of a harmful immune response to the phage itself. Compared to diluent treatment (supernatant from an overnight USA300 culture that had been pelleted and filter-sterilized in an identical manner to the phage cultures), exposing human peripheral mononuclear blood cells (PBMC) to increasing doses of phage did not induce pro-inflammatory responses as measured by IL-1ß, IL-6, IL-17A, or IFN gamma (Fig 4A–4D). To test if phage could induce inflammatory responses from keratinocytes, we co-cultured SATA-8505 with human keratinocytes (KC) derived from either primary foreskins (FSKC) or from the HaCaT cell line. Compared to diluent treatment, phage did not induce either IL-1ß or IL-6, but did induce small but statistically significant increases in IFN gamma in the FSKC cells (Fig 4E–4G). Of note, cells exposed to fresh broth had minimal cytokine induction, indicating stimulation by USA300-derived products in our diluent controls. Since primary human KC cultures can contain dendritic cell contamination, we also exposed the HaCaT cell line to phage and found no significant increase in IFN gamma (Fig 4G). This discrepancy may reflect dendritic cell contamination, or may be a reflection of the differences in HaCaT cell lines from primary cells [28,29].

### SATA-8505 Does Not Impact USA300 Growth in Human Blood

**Fig 5:**
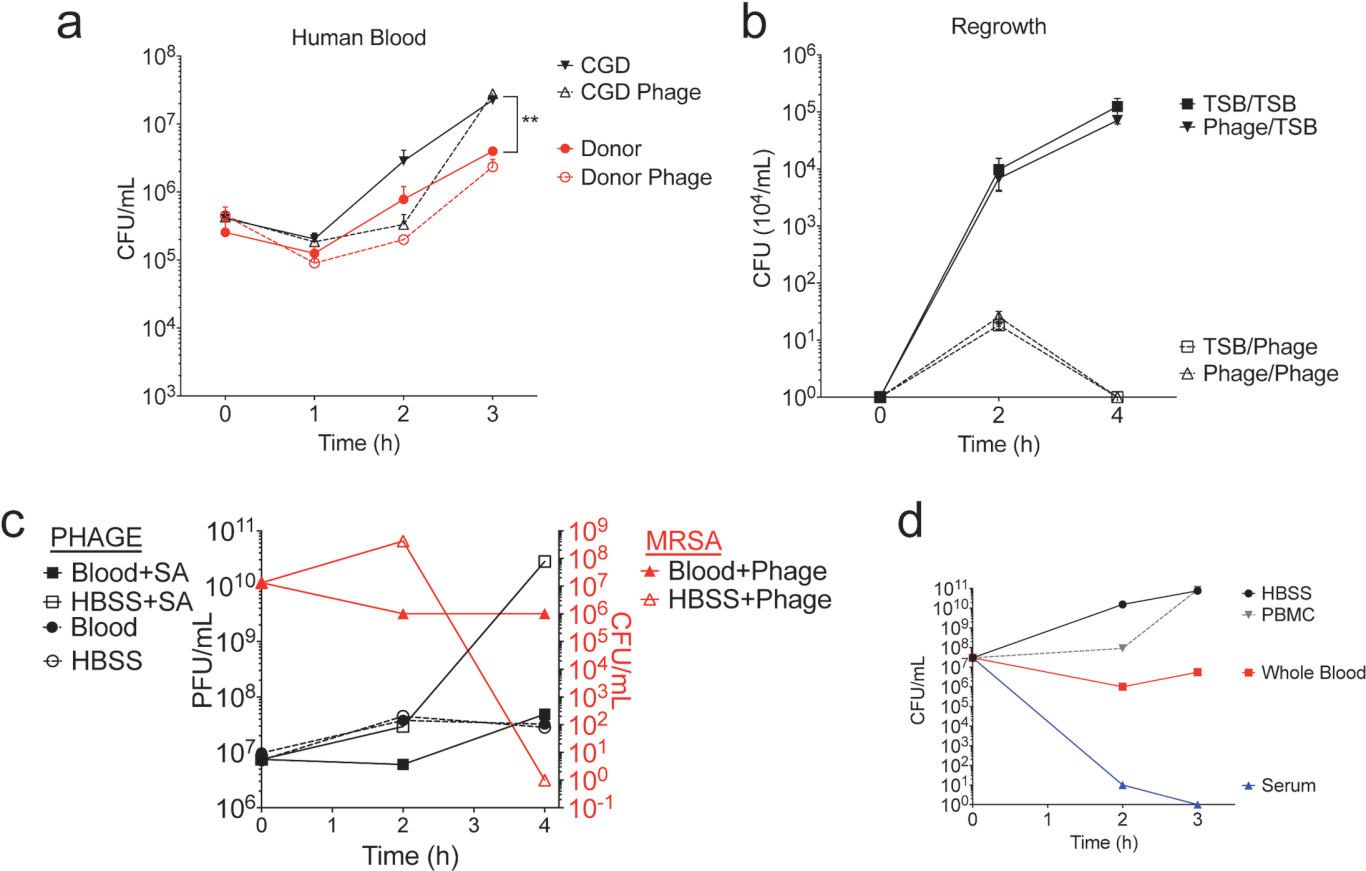
SATA-8505 Does Not Impact USA300 Growth in Human Blood. (a) Hourly quantification of starting culture of 10^6^ CFU USA300 in three parts TSB and one part whole blood from healthy donor or a patient with CGD, grown with or without 10^8^ PFU SATA-8505 in duplicate. (b) Regrowth of surviving colonies from whole blood culture run in triplicate. One colony of *S*. *aureus* previously grown in 3:1 TSB:Whole blood without phage was subsequently grown in TSB with SATA-8505 (TSB/Phage) or without phage (TSB/TSB); one colony of the surviving *S*. *aureus* previously grown in the presence of SATA-8505 was subsequently grown in TSB with SATA-8505 (Phage/Phage) or without phage (Phage/TSB). (c) Average quantification of starting culture of 10^10^ CFU of USA300 grown in either 3:1 TSB:Whole blood or 3:1 TSB:HBSS, with or without 10^7^ PFU SATA-8505. (d) Average quantification of starting culture of 10^7^ CFU of USA300 grown in three parts TSB with either one part whole blood, HBSS, serum, or peripheral mononuclear cells (PBMC) in equivalent volume HBSS. Data shown are representative of 2–3 independent experiments using 3 or more different healthy volunteers and 2 patients with CGD. Data is displayed as mean + s.e.m. ** = p<0.01.

To determine the effect of phage on bacterial growth in human blood, we inoculated blood from a healthy donor and a patient with CGD with 10^6^ CFU of USA300 and 10^8^ PFU of phage, monitoring bacterial growth in clinical blood culture bottles for three hours. As expected, CGD patients had greater bacterial growth in their blood than healthy volunteers. However, unlike the bactericidal effects seen in TSB media (Fig 1), phage did not significantly affect bacterial growth in blood (Fig 5A) or affect bacterial colony morphology (not shown). Bacteria exposed to phage in blood and then re-cultured in TSB grew just as well as bacteria unexposed to phage, and the bacteria were killed effectively upon a second exposure to phage in TSB (Fig 5B). These results indicated that the failure of phage to clear MRSA from the blood culture was not due to selection of a phage-resistant bacterium, and that the surviving colonies did not harbor phage the way they appeared to when re-cultured after being exposed to phage while grown in TSB (Fig 1C).

We next hypothesized that the failure of phage to clear a blood culture containing MRSA was due to direct clearance of the phage by the blood cells. However, we found no significant change in phage concentration when grown in blood, irrespective of the presence of *S*. *aureus* (Fig 5C; left y-axis). Again, phage did not significantly reduce MRSA colonies when grown in human blood, despite clearance of MRSA from culture media without blood (Fig 5C; right y-axis). Therefore, it appeared that the presence of human blood significantly reduced both MRSA and phage proliferation. We next separated whole blood into its cellular and serum components and again cultured USA300 in each of these for three hours. We found that at equal volumes, whole blood provided a bacteriostatic growth environment, PBMCs had no impact on growth, and serum had a bactericidal effect (Fig 5D). Given the mortality of MRSA bacteremia [30–32], it is clear that *S*. *aureus* does proliferate *in vivo*, however our results suggest that perhaps this growth occurs in seeded tissues rather than in the blood compartment directly. Our findings indicate that SATA-8505 phage treatment of USA300 bacteremia may not be effective at directly clearing the blood. This may reflect alterations in blood-borne USA300 that affect phage uptake and propogation, or may indicate the presence of inhibitory serum factors, such as albumin that may bind cations required for phage adsorption [33,34]. However, our results in the skin infection model as well as prior work showing protection from IV challenge suggest that phage therapy can control MRSA *in vivo*. Further investigation is needed to elucidate how SATA-8505 and other phages control MRSA pathology *in vivo* and to uncover within which compartments these benefits occur.

### SATA-8505 Exhibits Commercial Viability but Significant Strain Limitations

**Fig 6:**
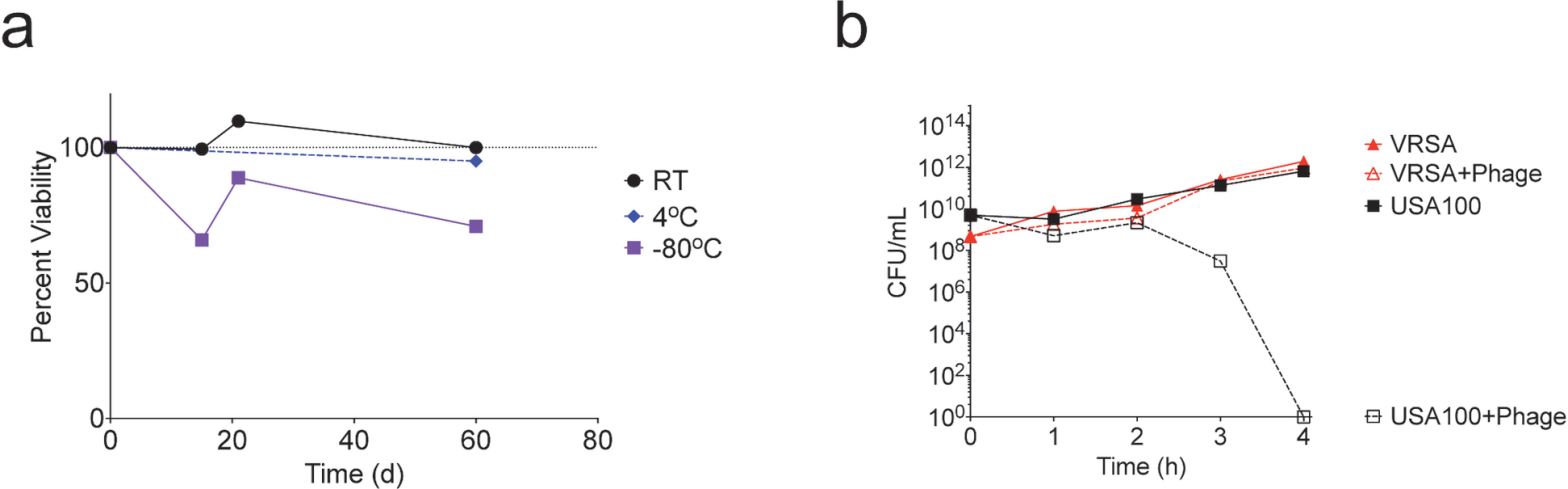
SATA-8505 Exhibits Commercial Viability but Significant Strain Limitations. (a) SATA-8505 quantified over up to sixty days after storage at either room temperature (RT), 4 degrees Celsius (4^°^C), or frozen at -80^°^C. (b) Average hourly quantification of 10^8^–10^9^ CFU of USA100 *S*. *aureus* or a vancomycin resistant variant of USA100 (VRSA) cultured with up to 10^11^ PFU of SATA-8505. Data shown are a representative of 2 independent experiments.

For a therapeutic phage to be clinically useful and a viable alternative to conventional antimicrobials, it must be easily stored and shelf stable, as pharmacies could not realistically be expected to culture batches de novo. To evaluate phage viability, we stored phage in frozen, refrigerated, and room temperature conditions and quantified PFU’s intermittently for 60 days. Phage was most stable at room temperature, and nearly 100% viable after 60 days (Fig 6A). Commercial labs have successfully lyophilized phage, suggesting lyophilization may be an option to further extend shelf life.

Another real-world challenge to therapeutic use of phages is their bacterial strain specificity. While strain-specificity may be useful for avoiding the unintended impacts on the microbiome seen with standard antibiotic treatment [35–40], it also limits the practical application of phage therapy, as the time needed to identify the causative strain would prohibit its use as a first-line treatment for acutely infected patients. Many strains of bacteria acquire resistance to antibiotics but remain otherwise similarly pathogenic, such as vancomycin resistant *Staphylococcus aureus* (VRSA). A VRSA strain on the USA300 background was not readily available, so we repeated the broth inoculation described using the MRSA USA100 strain 626 and a VRSA strain on the USA100 background (VRS8). While SATA-8505 cleared MRSA 626 from the broth culture, it did not have an impact on the growth of VRSA (Fig 6B). Three rounds of serial passage of phage through the VRSA culture to allow selection of potential VRSA-specific mutants did not enhance the killing ability (not shown). Although both MRSA 626 and VRS8 are pulsed-field type USA100 with matching spa type (Ridom t002) and multi-locus sequence type (ST 5), they are not isogenic strains. Thus, the difference in phage susceptibility may reflect vanA-mediated alterations in peptidoglycan structure in the VRSA strain [41], but may also reflect other differences between these two clinical isolates that will need further elucidation [42]. This difference in susceptibility, however, does highlight that relatively subtle alterations can render bacteria resistant to phage and may provide a platform for future mechanistic insights.

## Discussion

Our results confirm that SATA-8505 reduced viability of USA300, and found that it effectively controlled USA300 infection in human cells *in vitro* and in a mouse model of skin infection. This effect was *S*. *aureus* strain-specific and not observed in whole blood, where USA300 failed to proliferate and thus was not a viable phage target. SATA-8505 remained viable when stored at room temperature for two months. These results highlight the potential viability of phage therapy if limitations based on site of infection and strain of infecting bacteria can be overcome. Our results thus demonstrate both the promising attributes of phage therapy against USA300, and factors that may limit its real-world potential.

SATA-8505 is effective at killing USA300 and USA100 bacteria in standard growth conditions, can improve outcomes in MRSA skin infection when dosed appropriately, does not induce inflammatory responses in human PBMCs, and can be stored long-term without the need for refrigeration. However, we also found that SATA-8505 may induce inflammation in both mouse and human skin, could worsen MRSA skin infection or induce inflammatory damage in the immunocompromised if the dosing is overly aggressive, and has reduced clearance in mice with CGD. Additionally, SATA-8505 does not appear to kill USA300 when grown in the presence of human blood, and is incapable of killing our selected strain of VRSA even when on an otherwise susceptible USA100 background.

Our results also highlight the potential importance of appropriate dosing if phage therapy is used as a future therapeutic option. Although not an insurmountable limitation, most phage strains are incapable of infecting mammalian tissue and are thus rapidly cleared from the host [43,44]. Thus the timing of phage treatment must also be examined. Our choice to treat mice with phage immediately before infection was done to assure phage titers would be present at time of *S*. *aureus* exposure. Future work will be needed to see the time course of protection in this system as this timing may greatly alter the clinical utility phage as either a *S*. *aureus* therapeutic or prophylactic (for example, in patients on dialysis or patients that are post-operative). However, just because bacteriophage is not infectious to humans does not mean it does not harbor immunogenic potential, which may itself have detrimental effects on health. Like other biologic pharmaceuticals, higher doses may increase treatment effectiveness, yet do so at the risk of increasing treatment-induced complications [44]. More research is required to determine an optimal dosage that maximizes bacterial clearance while minimizing collateral damage to the host. Our results indicate that dosing may be of particular concern with immune compromised patients, as the higher dose of bacteriophage worsened infectious outcomes in our immunodeficient mice. Given that certain immune compromised individuals are more likely to suffer from MRSA infections, and would thus be the most likely candidates for phage therapy [4], researchers should be cautious of extrapolating dosing from early trials that are typically limited to healthy volunteers.

An additional consideration in assessing the potential utility of bacteriophage therapy is the issue of bacterial strain-specificity. While strain-specificity is useful for minimizing side effects of treatment on the patient microbiome, it also means that the exact bacterial strain will have to be known before phage therapy can be administered. Because this is unlikely to be feasible, the most promising option would be to administer a cocktail of several phage strains, similar to multivalent vaccines. Treating with several bacteriophages at once increases the likelihood of having a phage specific to the infective strain and thus increases the success rate of the therapy [45–49]. Furthermore, our work indicates that researchers may need to consider site of infection when formulating phage treatment, given that bacterial growth patterns may be altered by changes in the in vivo compartment in ways that may preclude therapeutic benefit.

There are many potential limitations to phage therapy becoming part of routine infection control. Our findings highlight the potential efficacy of phage therapy but also outline its scientific and therapeutic complexities such as dosing, host immune status, and site of infection. However, other notable limitations are of the regulatory and financial nature. For example, although cocktail treatment appear the best scientific model for the future, under current regulations each bacteriophage included in a proposed cocktail would have to undergo individual testing and show safety and effectiveness as an isolated therapy [49,50]. As discussed above, the strain-specificity of bacteriophage would likely render such testing unsuccessful; while each phage could be assessed for safety separately, each phage could only be expected to work in the fraction of cases that are caused by its target strain. Though the effectiveness of each individual phage may be low, the effectiveness of multiple phages may be additive [49]. This additive benefit will not be captured unless this treatment is allowed to undergo testing as a combination therapy. There are polyvalent Staphylococcal phages identified that could mitigate the need for combination therapy and SATA-8505 possesses lytic activity against 10–38% of the 60 strains of SA tested for the patent application [11]. However, our results suggest that even relatively minor genetic changes could greatly reduce treatment efficacy as seen in the susceptibility differences between two USA100 strains, MRSA 626 and VRS8. Therefore, phage therapy may benefit from being regulated in manners more similar to vaccines than chemical pharmaceuticals, where multivalent vaccines are not expected to show efficacy of each component in isolation and polyvalent phages could be combined for greater coverage through redundancy of strain targets.

When phage is applied to food products for the purposes of controlling bacterial growth, it is deemed “generally regarded as safe” by the Food and Drug Administration and thus requires no pre-market proof of safety, as exemplified by the use of phage in cheese to protect against the growth of *Listeria monocytogenes* [49]. While such loose regulatory ideals are inappropriate for pharmaceuticals, it is not inconceivable that regulations on phage therapy could be amended to reflect the current scientific understanding. Other notable regulations proposed for safe clinical use of phages use include the availability of the full phage genome sequence, verification of the lack of toxin producing elements, lack of lysogenic potential, evaluation of transducing abilities, production under Good Manufacturing Practices, and (if possible) propagation in a non-pathogenic strain of bacteria [51]. The sequence of SATA-8505 is available and reveals the phage to be from the Myoviridae family, but the remaining details would need to be verified before therapeutic consideration. Accordingly, future work using Myoviridae phage therapies should consider the potential to induce inflammation in human keratinocytes.

Even with regulatory reforms, there would still be concerns related to financial incentives to produce phage therapy. Bacteriophage can be easily produced using basic materials, and could be readily cultured outside of a pharmaceutical laboratory. A second supply stream would threaten the financial benefits of legal production, and may be difficult to control in parts of the world where drug production is not vigilantly regulated. Such a phenomenon is a concern with all pharmaceuticals, but is of particular interest with phage because of this ease of replication. Furthermore, products of nature are not open to being patented, which was upheld as recently as 2013 in the United States Supreme Court case *Association for Molecular Pathology v*. *Myriad Genetics*. The seemingly reasonable basis for such patent restrictions, along with the potential for ‘biologic piracy’ mentioned before, would suggest that profit-motives are unlikely to drive phage development and thus government officials would need to be proactive both for regulatory changes and phage development on both a national and international scale.

However, despite all of these limitations, another conclusion that must be drawn from the literature is that the response to multi-drug resistant bacterial infections must be a top priority. Beyond the threat of a “post-antibiotic era”, the newly elucidated potential for antibiotic-induced harm to the normal microbiome [35] has further highlighted the need for new approaches when developing targeted anti-pathogen treatments. Urgent facilitation of research into human dosing, efficacy, and best regulatory practices for phage therapy could assure a more timely response if any high-threat, multidrug resistant pathogen should become an emergent pandemic [52]. Ultimately, in spite of significant logistical challenges faced by phage therapy, this type of research highlights an important direction for science. As both antibiotic resistance and immune compromised populations continue to increase, the need for alternative therapies, and alternative therapeutic mechanisms, is urgent. As natural predators of bacteria, bacteriophages remain a promising option for controlling bacterial infections. However, future research, and perhaps refinement of the legal process of drug development, are needed before this may become a reality.
